# The formation of tau pore-like structures is prevalent and cell specific: possible implications for the disease phenotypes

**DOI:** 10.1186/2051-5960-2-56

**Published:** 2014-05-29

**Authors:** Cristian A Lasagna-Reeves, Urmi Sengupta, Diana Castillo-Carranza, Julia E Gerson, Marcos Guerrero-Munoz, Juan C Troncoso, George R Jackson, Rakez Kayed

**Affiliations:** Mitchell Center for Neurodegenerative Diseases, University of Texas Medical Branch, Galveston, TX USA; Departments of Neurology and Neuroscience & Cell Biology, University of Texas Medical Branch, 301 University Blvd. Medical Research Building, Room 10.138C, Galveston, TX 77555 USA; Division of Neuropathology, Department of Pathology, Johns Hopkins University School of Medicine, Baltimore, MD USA; Department of Molecular and Human Genetics, Baylor College of Medicine, Jan and Dan Duncan Neurological Research Institute at Texas Children’s Hospital, Houston, TX 77030 USA

**Keywords:** Tau, Annular protofibrils, Oligomers, Tauopathies

## Abstract

Pathological aggregation of the microtubule-associated protein tau and subsequent accumulation of neurofibrillary tangles (NFTs) or other tau-containing inclusions are defining histopathological features of many neurodegenerative diseases, which are collectively known as tauopathies. Due to conflicting results regarding a correlation between the presence of NFTs and disease progression, the mechanism linking pathological tau aggregation with cell death is poorly understood. An emerging view is that NFTs are not the toxic entity in tauopathies; rather, tau intermediates between monomers and NFTs are pathogenic. Several proteins associated with neurodegenerative diseases, such as β-amyloid (Aβ) and α-synuclein, have the tendency to form pore-like amyloid structures (annular protofibrils, APFs) that mimic the membrane-disrupting properties of pore-forming protein toxins. The present study examined the similarities of tau APFs with other tau amyloid species and showed for the first time the presence of tau APFs in brain tissue from patients with progressive supranuclear palsy (PSP) and dementia with Lewy bodies (DLB), as well as in the P301L mouse model, which overexpresses mutated tau. Furthermore, we found that APFs are preceded by tau oligomers and do not go on to form NFTs, evading fibrillar fate. Collectively, our results demonstrate that in vivo APF formation depends on mutations in tau, phosphorylation levels, and cell type. These findings establish the pathological significance of tau APFs in vivo and highlight their suitability as therapeutic targets for several neurodegenerative tauopathies.

**Electronic supplementary material:**

The online version of this article (doi:10.1186/2051-5960-2-56) contains supplementary material, which is available to authorized users.

## Introduction

The microtubule-associated protein tau plays important cellular roles, including regulating microtubule assembly and stability, axonal transport, and neurite outgrowth [1]. Most of the biological functions of tau are modulated by site-specific phosphorylation [2]. Tau self-assembly, aggregation, and the accumulation of neurofibrillary tangles (NFTs) are hallmarks of Alzheimer’s disease (AD) and other neurodegenerative conditions [3, 4]. Although the importance of tau in AD and other tauopathies is well established [5–7], it is unclear whether NFTs are a primary neurotoxic agent. Most research has focused on NFTs because of the reported correlation between NFT levels in AD brain and disease progression [8–10]; however, recent data suggest that soluble pre-filamentous tau species may be the most toxic and pathologically significant tau aggregates [11, 12]. For instance, cell death and synaptic lesions occur independently of NFT formation in htau mice expressing non-mutant human tau [13, 14]. Furthermore, hippocampal synapse loss and microgliosis precede NFT formation in the P301S transgenic mouse model (P301S Tg), which overexpresses mutated tau [15]. Moreover, in a conditional mouse model (rTg4510) expressing the P301L htau mutant, soluble tau level was found to correlate with neuronal loss and behavioral deficits to a greater degree than NFT level [16, 17].

Soluble oligomers have been implicated as the primary toxic species in many degenerative diseases in which the accumulation of large fibrillar deposits may be inert, protective, or pathological by a different mechanism [18, 19]. However, their structures, interrelationships with other amyloid aggregates, and exact contribution to disease pathogenesis are not entirely clear [20–23]. Amyloid-β (Aβ), α-synuclein, and other amyloidogenic proteins form annular protofibrils (APFs) in vitro; these pore-like structures have been observed in synthetic preparations of both oligomers and fibrils [24, 25]. The formation of pores by Aβ and α-synuclein aggregates are accelerated when the APFs are generated using proteins with mutations associated with familial Alzheimer’s and Parkinson’s diseases, respectively, suggesting that these mutated proteins are more prone to pathogenic activity [26]. More importantly, in addition to the generation of these structures from synthetic peptides, APFs have been isolated from inclusions in the brains of patients with multiple system atrophy (MSA) [27] and AD [28, 29]. Electron microscopy (EM) and atomic force microscopy (AFM) of these structures revealed that they vary in shape and size, lending support to the concept of nonspecific pore formation and membrane leakage. The amyloid pore hypothesis [30] suggests that similar to bacterial pore-forming toxins [31–33], amyloid oligomers/protofibrils induce cell death by disrupting regulated membrane permeability, which disrupts cellular ion and protein homeostasis. Membrane permeabilization is a common pathogenic effect of amyloid oligomers [34], which are APF precursors. The off pathway conversion of oligomers to APFs, which penetrate the membrane, is an attractive explanation for this toxic effect given the shared assembly state and morphological resemblance between APFs and bacterial pores.

Despite overwhelming evidence supporting amyloid pore formation from biochemical, biophysical, and cell culture experiments [26], tau pore formation has not been shown in vitro or in vivo. The purpose of the present study was to determine the relationship of tau APFs with other tau amyloid species, such as small oligomers, and to determine if tau APFs are found in human brain samples and the P301L Tg mouse model. Using the well-characterized conformational antibody αAPF, which specifically detects APFs independently of amino acid sequence [28, 29, 31, 35], we found that APFs are preceded by tau oligomers, are on a distinct pathway from NFT formation, and that these pore-like structures are present in human and P301L Tg mouse brain tissue. Furthermore, our results indicate that in vivo APF formation depends on tau phosphorylation, the presence of mutations, and cell type. These findings demonstrate the pathological significance of tau APFs for several neurodegenerative tauopathies and highlight their suitability as therapeutic targets for these diseases.

## Materials and methods

### Preparation of Tau oligomers

Recombinant tau protein (tau-441 [2N4R], MW 45.9 kDa) was expressed and purified as described previously [1, 36]. It was treated with 8 M urea to obtain monomeric tau; then it was dialyzed overnight against 1× PBS buffer (pH 7.4) and adjusted to 1 mg/mL with PBS, and aliquots of tau monomer (in PBS) were stored at -20°C. For oligomer preparation, 300 μL tau stock (1 mg/mL) was added to 700 μL 1× PBS, for a final concentration 0.3 mg/mL. Aβ42 oligomers (7 μL, 0.3 mg/mL) were added to the sample (seeds) and mixed by pipetting for 1 min. The sample was then incubated at room temperature for 1 h on an orbital shaker, and the resulting tau oligomers were used to seed a new batch of tau. This procedure was repeated three times to eliminate the residual Aβ seeds. The preparation and characterization of tau oligomers were performed as described previously [37, 38]. Paired helical filament (PHF) tau fibrils from full-length recombinant tau protein were prepared using heparin according to well-established protocols [1, 36].

### Preparation and characterization of Tau APFs

A homogeneous population of pore-like tau APFs was generated by using tau oligomers as the starting material. First, 5% (v/v) hexane was added to a solution of oligomers, and the sample was mixed with a vortex mixer for 1 min every 5 min for a total of 50 min. Then the samples were dialyzed in water, using a molecular mass cut-off membrane of 10 kDa. The morphologies of tau APF preparations were characterized by EM and AFM and purified by size-exclusion chromatography using an LC-6 AD Shimadzu high-performance liquid chromatography (HPLC) system (Shimadzu, Kyoto, Japan) fitted with a Sephacryl S-500 HR (10 × 600 mm) column, (cat number: 17-0613-10; GE Healthcare Life Sciences, Little Chalfont, UK). PBS (pH 7.4) was used as the mobile phase at a 1.5 mL/min flow rate. A gel filtration standard (51–1901; Bio-Rad, Hercules, CA) was used for calibrations.

### EM

For each sample, 2 μL was adsorbed onto 200-mesh carbon and Formvar-coated grids, air-dried, and washed for 1 min in distilled water. The samples were negatively stained with 2% uranyl acetate (Ted Pella Inc., Redding, CA) for 2 min and viewed with a Zeiss 10CR microscope (80 kV; Carl Zeiss, Oberkochen, Germany).

### AFM

The morphologies of synthetic tau APFs and immunoprecipitated structures was assessed by AFM by a non-contact tapping method (ScanAsyst-Air, Bruker, Billerica, MA) using a Multimode 8 AFM machine (Veeco, Plainview, NY).

### Liposome preparation

For liposome preparation, 10 mg phosphatidylcholines (Sigma, St. Louis, MO) was dissolved in 500 μL chloroform (20 mg/mL). The chloroform was evaporated under a stream of nitrogen in the hood, the resulting film was re-hydrated with 500 μL buffer (10 mM HEPES, 100 mM NaCl, pH 7.4), and the solution was intensely vortexed for 3–5 min. Tau oligomers were dissolved to 66 μM in H_2_O and incubated with liposomes in PBS (1/10 [v/v] liposome/Tau oligomers) for 2 h at room temperature.

### Human brain extracts

All postmortem brain tissue used were randomized. Tissue from patients with progressive supranuclear palsy (PSP) and control subjects were provided in frozen blocks by the Brain Resource Center at Johns Hopkins University (Baltimore, MD). Tissue cases were collected with patient consent and handled under protocols approved by the Johns Hopkins Institutional Review Board. PSP samples were examined at the Division of Neuropathology at John Hopkins University. For this study, PSP tissue from the pons was utilized. The dementia with Lewy Bodies (DLB) cases were obtained from the Oregon Brain Bank at Oregon Health and Science University (OHSU, Portland, OR). Tissue use conformed to OHSU Institutional Review Board approved protocols. Neuropathologic assessment conformed to National Institute on Aging-Reagan consensus criteria.

Donor subjects were enrolled and clinically evaluated in studies at the NIH-sponsored Layton Aging and AD Center (ADC) at OHSU. Subjects were participants in brain aging studies at the ADC and received annual neurological and neuropsychological evaluations, with a clinical dementia rating (CDR) assigned by an experienced clinician. Cortex from DLB cases was used in the present study. Each brain tissue sample was homogenized in PBS with a protease inhibitor cocktail (catalog no. 11836145001; Roche Applied Science, Indianapolis, IN), using a dilution of brain: PBS of 1:3 (w/v). Samples were then centrifuged at 10,000 rpm for 10 min at 4°C. The supernatants were portioned into aliquots, snap-frozen, and stored at -80°C until use [28].

### Transgenic mouse model

P301L (JNPL3) mice (Taconic Farms) were used for our experiments, including biochemical and immunohistochemistry (IHC) analyses. The JNPL3 mouse model [39] expresses a frontotemporal lobar degeneration-associated human mutant tau transgene (P301L). Mice were housed at the University of Texas Medical Branch (UTMB) animal care facility and were maintained according to USDA standards (12-h light/dark cycle, food and water ad libitum), in accordance with the Guide for the Care and Use of Laboratory Animals (National Institutes of Health, Bethesda, MD). Animals were anesthetized according to UTMB Institutional Animal Care and Use Committee-approved procedures. Tissue was collected from 2-, 4-, 7-, 10-, and 12-month-old animals. Prior to euthanasia, mice were deeply anesthetized and perfused transcardially with 1× PBS prior to decapitation. Animals were anesthetized according to IACUC-approved procedures for animal sacrifice. After sacrifice, brains and spinal cords were dissected and stored at -80°C. Mouse tissue was homogenized as previously described for human samples [40].

### Immunoprecipitation of APFs from human brain tissue

The characterization and specificity of the αAPF conformation-specific antibody was published previously [31]. For immunoprecipitation experiments, tosyl-activated magnetic Dynabeads (Dynal Biotech, Lafayette Hill, PA) were coated with 11.25 μg αAPF antibody (2.6 mg/mL) and diluted in 0.1 M borate (pH 9.5) overnight at 37°C. Beads were washed (0.2 M Tris, 0.1% bovine serum albumin [BSA], pH 8.5) and then incubated with either DLB or control patient brain homogenate with rotation at RT for 1 h. Beads were then washed three times with PBS and eluted using 0.1 M glycine (pH 2.8). The pH of each eluted fraction was adjusted using to pH 8.0 with 1 M Tris. Finally, some fractions were applied to a nitrocellulose membrane for dot blotting with the Tau-5 antibody (Covance, Princeton, NJ). Other fractions were again co-immunoprecipitated using spin columns (Cat# 26149, Thermo Scientific, Waltham, MA) coated with Tau-5 antibody to ensure the presence of tau APFs for subsequent AFM imaging.

### Dot blot

Each sample (2 μl) was applied to a nitrocellulose membrane, blocked with 10% nonfat milk in Tris-buffered saline with Tween (TBS-T) overnight at 4°C, washed three times for 5 min each with TBS-T, and incubated for 1 h at RT with the anti-APF antibody αAPF (1:1000) for the synthetic samples and the anti-tau antibody Tau-5 (1:5000) for the immunoprecipitated samples. The membranes were washed three times for 5 min each with TBS-T, incubated with horseradish peroxidase (HRP)-conjugated anti-rabbit IgG (Promega, Madison, WI) diluted 1:10000 in 3% BSA/TBS-T, and incubated for 1 h at room temperature. The blots were washed 3 times with TBS-T and developed with an enhanced chemiluminescence kit from (RPN2132; Amersham-Pharmacia/GE Healthcare Life Sciences, Little Chalfont, UK).

### Immunofluorescence

Paraffin-embedded brain sections from DLB, PSP, and control subjects and JNPL3 mice were hydrated using xylene, 100% ethanol, 95% ethanol, 80% ethanol, and distilled water. Then, the sections were heated by microwave (750 watts) in target solution (Dako, Glostrup, Denmark) twice for 4 min each. The tissues were washed in 1× PBS, three times for 5 min each, and blocked for 45 min with 5% horse serum in PBS. The tissues were incubated with rabbit anti-APF antibody αAPF antibody (1:350) overnight. Then the sections were washed three times with 1× PBS for 10 min each. The sections were incubated with the secondary antibody anti-rabbit Alexa Fluor 568 (1:700; Invitrogen, Carlsbad, CA) for 60 min. Then, the sections were washed three times with 1× PBS for 5 min each and blocked again with 5% horse serum in PBS for 30 min. The sections were then washed three times for 10 min each time in PBS before incubation overnight with the following antibodies: mouse anti-tau Tau-5 (1:1000), anti-phospho tau (Ser202/Thr205) AT8 (1:1000, Thermo Fisher), glial fibrillary acid protein (GFAP) (1:5000, Covance), NeuN (1:3000, Chemicon International Inc., Temecula, CA), myelin oligodendrocyte glycoprotein (MOG) (1:800; LifeSpan Biosciences, Seattle, WA) anti-Aβ 4G8 (1:800, Covance), and anti-α-synuclein 4D6 (1:700, Covance). The next day, the sections were washed three times in PBS for 10 min each before incubation with goat anti-mouse IgG Alexa Fluor 488 (1:700, Invitrogen) for 1 h. Sections were washed and mounted in Vectashield mounting medium with 4′,6-diamidino-2-phenylindole (DAPI; Vector Laboratories, Burlingame, CA). The sections were examined using a Bio-Rad Radiance 2100 confocal system (Bio-Rad, Hercules, CA) mounted on a Nikon Eclipse E800 microscope (Nikon, Tokyo, Japan) equipped with a CoolSnap-FX monochrome CCD camera (Photometrics, Tucson, AZ) using a standard Nikon FITC, TX Red, and DAPI filter set for Alexa Fluor 488 and 568 and DAPI, respectively.

### APF IHC

Paraffin-embedded brain tissue sections from DLB, PSP, and control subjects and JNPL3 mice were hydrated using xylene, 100% ethanol, 95% ethanol, 80% ethanol, and distilled water. The hydrated sections were heated twice for 4 min each in target solution (Dako) using a microwave oven (750 watts). Tissues were then washed in 1× PBS three times for 5 min each and blocked for 10 min with 3% H_2_O_2_ in PBS. Tissues were then washed in 1× PBS three times for 5 min each and blocked for 60 min with 5% horse serum in PBS. The tissues were incubated with αAPF antibody (1:350) overnight. The sections were then washed three times with 1× PBS for 10 min each, followed by a 60-min incubation with the secondary biotinylated anti-rabbit antibody (Pierce Biotechnology, Rockford, IL). The sections were washed three times with 1× PBS for 5 min each. The sections were then incubated for 30 min using an ABC kit and washed with 1× PBS three times for 5 min each. Finally, the sections were incubated with 3,3′-diaminobenzidine (DAB) for 5 min.

### Tau APF formation induced by myelin basic protein (MBP)

Purified human MBP (Chemicon International, Temecula, CA) was resuspended in 20 mM sodium acetate and 100 mM sodium chloride (pH 4.0), dialyzed into PBS, and stored at -80°C at 1 mg/mL. Tau oligomers (0.3 mg/mL) were incubated at 37°C with rocking, either alone or with 1.56 μM MBP or 1.56 μM bovine lactalbumin. Control samples containing Aβ oligomers and 1.56 μM MBP or 1.56 μM MBP in PBS were also included. At each time point, 100 μL samples of each reaction were placed in a 96-well microplate in triplicate, and the amount of APF formation was determined by enzyme-linked immunosorbent assay (ELISA) using the αAPF antibody.

### Direct ELISA

For ELISA, plates were coated with 100 μL sample using 0.1 M sodium bicarbonate (pH 9.6) as coating buffer, followed by overnight incubation at 4°C and three washes with TBS-T (0.01% Tween). Then, plates were blocked for 1 h at RT with 10% BSA (IgG-free TBS-T). The plates were then washed three times with TBS-T before the primary antibody (αAPF, 1:500; T22, 1:1000; Tau5 1:1000) was added and allowed to react for 1 h at RT. The plates were then washed three times with TBS-T, and 100 μL HRP-conjugated anti-rabbit IgG or anti-mouse IgG (diluted 1:10,000 in 5% nonfat milk in TBS-T; Promega) was added, followed by incubation for 1 h at RT. Finally, plates were washed three times with TBS-T and developed with 3,3,5,5-tetramethylbenzidine (TMB-1 component substrate) from KPL (Gaithersburg, MD). The reaction was stopped with 100 μL 1 M HCl, and samples were read at 450 nm. Statistical analyses were based on a two-way analyses of variance (ANOVAs), performed using Origin-8 software (Origin Lab, Northampton, MA).

### Competitive ELISA

For competitive ELISA, plates were coated with 100 ng tau oligomer (for T22) or APF (for αAPF) using 0.1 M sodium bicarbonate (pH 9.6) as the coating buffer, followed by overnight incubation at 4°C. This protocol follows the same directions indicated for the direct ELISA with the exception that αAPF and T22 were pre-incubated with tau monomers, oligomers, APFs, or fibrils for 45 min. During the incubation, the amount of antibody was four times greater than that of each tau species. Six samples were run for each condition. For statistical analysis, two-way ANOVA was performed using Origin-8 software.

### Cell toxicity assay

Neuron viability was assessed using the MTT colorimetric assay (Invitrogen) as previously described [31]. Briefly, SH-SY5Y human neuroblastoma cells were treated with 2 μM tau oligomers, monomers, or APFs. After incubation for 6 h at 37°C, the cells were assayed using a 3-(4,5-dimethylthiazol-2-yl)-2,5-diphenyltetrazolium bromide (MTT) toxicity assay kit (Tox-1) (Sigma) according to the manufacturer’s directions. All measurements were made in triplicate. Statistical analyses were based on a two-way ANOVAs, which were performed using Origin-8 software.

### Immunocytochemistry

SH-SY5Y cells were treated with 2 μM tau oligomers or monomers for 6 h. Then, the cells on the coverslip were fixed and permeabilized. The samples were blocked for 1 h in 5% goat serum and incubated overnight at 4°C with Tau-5 (1:1000). Sections were then washed and incubated with an Alexa 488-conjugated goat anti-mouse antibody (1:700, Invitrogen) for 1 h at RT. Sections were then incubated overnight at 4°C with αAPF (1:350), then washed and incubated with an Alexa 568-conjugated goat anti-mouse antibody (1:700). DAPI was used to stain the nuclei (1:4000, Invitrogen). Sections were then washed for 30 min and coverslipped.

## Results

### Tau oligomers form APFs in vitro

**Figure 1:**
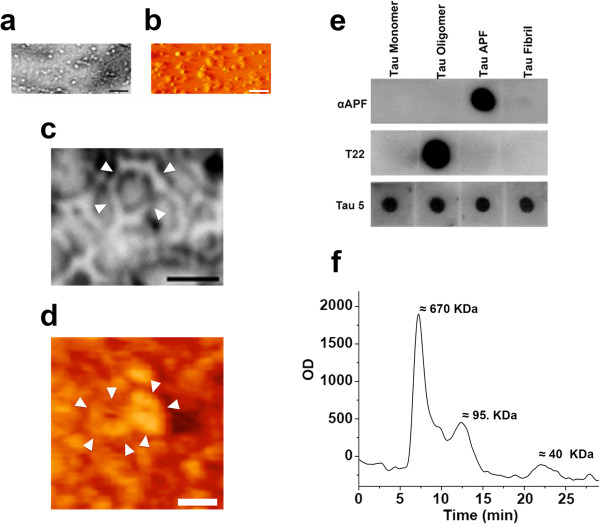
***In vitro*** **formation of tau APFs.** Tau oligomers used to prepare tau APFs were characterized by EM **(a)** and AFM **(b)**. Tau APFs were also imaged by EM **(c)** and AFM **(d)**. Tau APFs were recognized by the αAPF antibody on dot blot, which did not recognize recombinant tau monomers, oligomers, or fibrils. Tau oligomers were recognized by T22, and all tau species were detected by Tau-5 **(e)**. A distinct peak near 670 nm on HPLC revealed that tau APFs are formed by 10–12 tau monomers **(f)**. The scale bars for **a** and **b** correspond to 100 nm, for **c** 20 nm, and for **d** 40 nm. White arrowheads indicate tau APF formation.

To determine whether tau forms APFs similarly to other amyloid proteins, full-length human tau protein was used to prepare tau oligomers as previously described [37, 38]. The presence of tau oligomers was confirmed by both EM (Figure 1a) and AFM (Figure 1b) analysis. Using tau oligomers as the starting material, APFs were prepared with 5% hexane and characterized using EM (Figure 1c) and AFM (Figure 1d). As we previously demonstrated, liposomes can catalyze the conversion of oligomers to APFs under more physiologically relevant conditions than treatment with 5% hexane [31]. When liposomes were reconstituted with tau oligomers and incubated for 2 h, APFs were detected by AFM (Additional file 1: Figure S1a). The formation of tau APFs was also confirmed by dot blot using the APF-specific antibody, αAPF, which did not recognize tau monomers, oligomers, or fibrils (Figure 1e). The selectivity of αAPF for tau APFs was confirmed by competitive ELISA (Additional file 1: Figure S1b). The conformational tau antibody T22 was used to detect tau oligomers in dot-blot (Figure 1e). The selectivity of T22 for tau oligomers has been previously described [40]. Nevertheless, we confirmed its selectivity for the oligomeric form of tau by direct and competitive ELISAs (Additional file 1: Figure S1c and d). The HPLC analysis of tau APF prepared in vitro showed a peak over 670 KDa, suggesting that APFs range from decamers to dodecamers of tau monomers (Figure 1f). We compared the toxicity of tau APFs and oligomers and found that APFs are significantly less toxic (Additional file 1: Figure S1e). As we previously reported for Aβ and α-synuclein [31], tau APFs are less toxic than the oligomers used to prepared them. Moreover, whenever cells were treated with tau oligomers we observed the formation of tau APFs whenever cells were treated with tau oligomers, suggesting that one of the toxic mechanisms of tau oligomers is APF formation (Additional file 1: Figure S1f).

### Tau APFs are present in DLB and PSP brain tissue and are on a distinct pathway from hyperphosphorylated NFT formation

**Figure 2:**
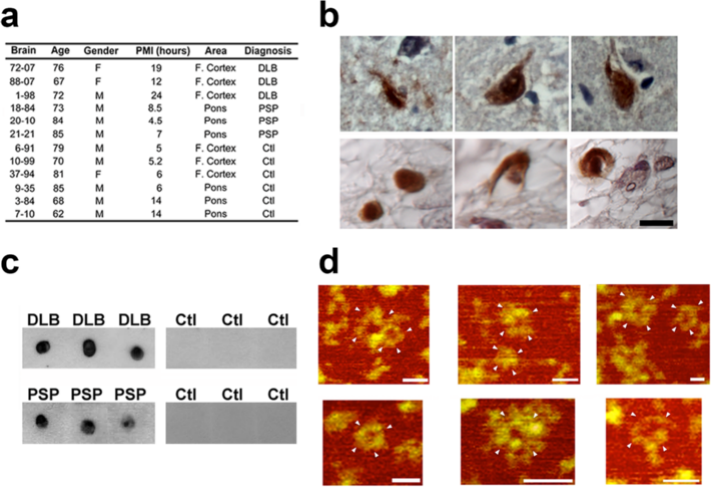
**Tau APFs are present in DLB and PSP brain tissue.** The age, gender, post-mortem interval (PMI), and sampled brain regions in each group is summarized **(a)**. APFs were detected in DLB cortex (top panel) and PSP pons (bottom panel) by light field microscopy after immunolabeling with αAPF **(b)**. After immunoprecipitation using αAPF, tau annular structures were detected in DLB and PSP cases but not in age-matched control brain tissue (top, cortex; bottom, pons) by dot-blot analysis using Tau-5 antibody **(c)**. Tau amyloid pores isolated from DLB (top) and PSP (bottom) brain tissue were visualized by AFM **(d)**. Scale bar for HRP staining, 10 μm. Scale bar for AFM images, 30 nm. White arrowheads indicate tau APFs.

The brain samples from patients with DLB and PSP and age-matched controls used in this study are described in Figure 2a. We analyzed DLB cases to study the presence of tau APFs because although α-synuclein aggregates are considered the primary pathological hallmark, a subset of cases also exhibit tau aggregation in several cell types, including neurons, astrocytes, and oligodendrocytes [41]. Similarly, PSP is mainly characterized by the deposition of tau aggregates in neurons, astrocytes, and oligodendrocytes [42]. Our analysis allowed us to determine which structural form of tau leads to APF formation. To investigate the presence of pore-like structures in human DLB and PSP brains, we used the αAPF antibody, which enabled us to distinguish APFs from other tau aggregates. IHC of paraffin-embedded sections using the αAPF antibody and DAB revealed strong immunolabeling in both DLB and PSP cases (Figure 2b). When IP was performed using αAPF and probed with Tau-5 by dot blot, the presence of tau APFs was seen in all DLB and PSP disease cases, with no reactivity seen in control brain samples (Figure 2c). Subsequent examination of isolated tau APFs by AFM revealed that these structures were similar to those prepared from synthetic peptides (Figure 2d). Additionally, αAPF immunoprecipitated from control tissues did not pull down any APFs detectable by AFM (data not shown). Immunoprecipitated APFs were more homogeneous in size (~15 nm in diameter) than synthetic APFs (~5-20 nm in diameter). Although the immunoprecipitated APFs were morphologically similar to the synthetic peptides, they were not detected with Aβ and α-synuclein antibodies (4G8 and 4D6, respectively) by dot blot (data not shown), which suggests that APFs in DLB brain are primarily composed of tau protein.

**Figure 3:**
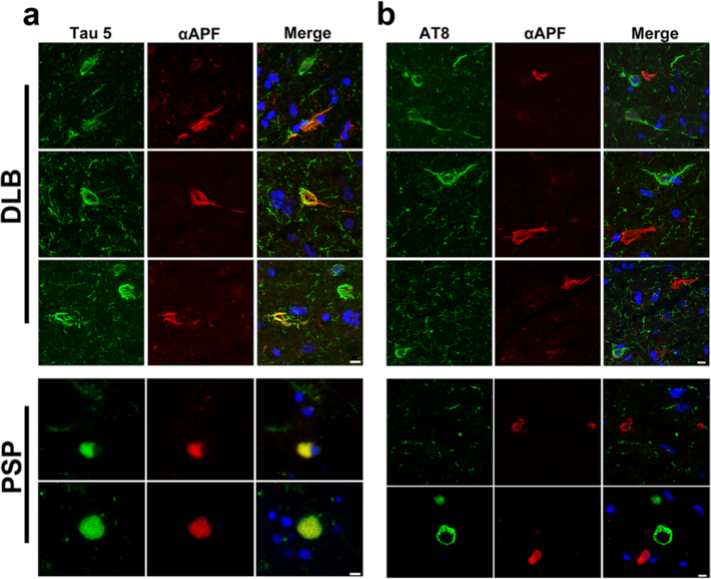
**Tau APFs are not phosphorylated at Ser202/Thr205. (a)** Tau APFs were examined after double immunofluorescence labeling using Tau-5 (green) and αAPF (red) in DLB and PSP sections. **(b)** Double labeling with AT8 (green) and αAPF (red) revealed lack of colocalization in all of cases, indicating that tau APFs are not largely phosphorylated at Ser202/Thr205. Scale bar, 10 μm.

To confirm that the APFs detected in DLB and PSP samples were indeed tau APFs, we performed double immunofluorescence labeling using the αAPF antibody and the tau-specific Tau-5 antibody. High levels of colocalization were seen in sections colabeled with αAPF and Tau-5, which recognizes all forms of tau (Figure 3a and Additional file 2: Figure S2a). However, in double labeling using AT8, which recognizes tau phosphorylated at Ser202/Thr205 (found in NFTs) [43], no colocalization was for AT8 and αAPF (Figure 3b and Additional file 2: Figure S2b). Therefore, it is likely that tau APFs are not highly phosphorylated at epitopes Ser202/Thr205 and arise from a different form of tau than that found in NFTs.

Because tau APFs are best prepared from tau oligomers in vitro, DLB brain sections were also analyzed for the presence of tau oligomers colocalized with APFs by immunofluorescent double labeling with αAPF and T22, a polyclonal conformation-specific antibody against tau oligomers [40]. Partial colocalization between tau oligomers and APFs was seen (Additional file 3: Figure S3), which is in agreement with the model suggesting that oligomers form initially and proceed on a separate APF formation pathway [28].

**Figure 4:**
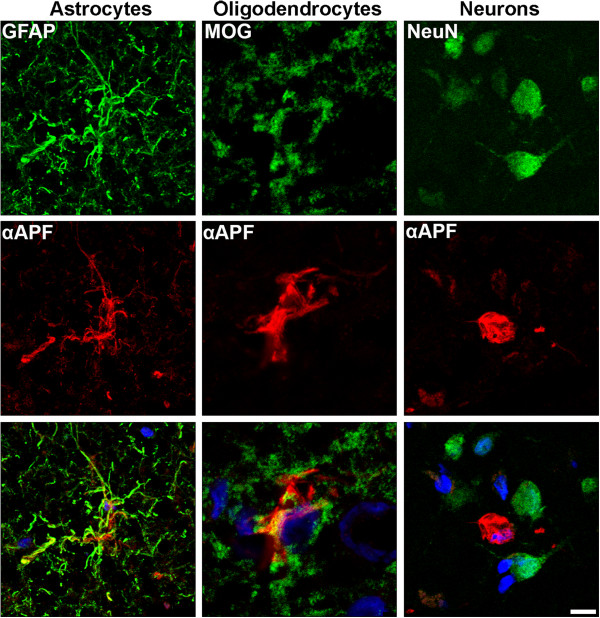
**Cell specificity of tau APFs in DLB.** Double labeling with GFAP (green) and αAPF (red) demonstrated the presence of tau APFs in astrocytes in DLB brains. Tau APFs in oligodendrocytes were demonstrated by double labeling with antibodies against MOG (green) and αAPF (red). Tau APFs were not detected in neurons in DLB brains as shown by double labeling using NeuN (green) and αAPF (red). Scale bar, 10 μm.

To determine whether APF formation is specific to certain cell types, DLB and PSP brain sections were examined using immunofluorescent labeling with αAPF and GFAP, MOG, or NeuN antibodies recognizing astrocytes, oligodendrocytes, and neurons, respectively. Figure 4 shows that tau APFs were found in oligodendrocytes and astrocytes in DLB brain, but not in neurons. Conversely, APFs were detected in all analyzed cell types in PSP brain (Additional file 4: Figure S4). We did not identify any tau pathology in the microglia in either the DLB or PSP tissue (data not shown). It is well-known that DLB is characterized by the presence of Lewy bodies and amyloid plaques composed of aggregated α-synuclein and Aβ, respectively. To determine if these amyloidogenic proteins also form APFs in DLB, we performed double labeling with the αAPF antibody and the anti-Aβ antibody 4G8 or the anti-α-synuclein antibody 4D6. We did not observe any colocalization between αAPF and 4G8 or 4D6, suggesting either that Aβ and α-synuclein do not form annular structures in DLB or that the epitopes recognize by 4G8 and 4D6 are not exposed in these structures (Additional file 5: Figure S5).

### Tau APFs are present in the JNPL3 mouse model

**Figure 5:**
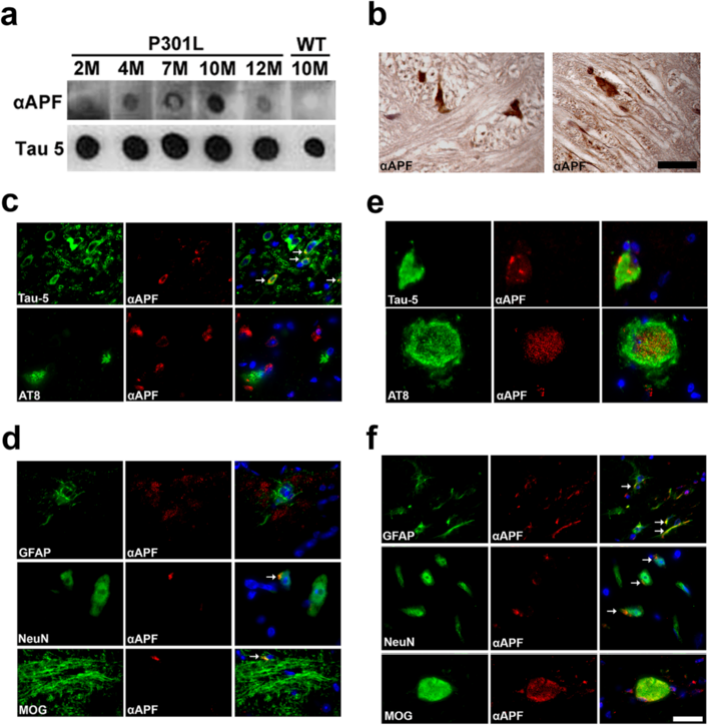
**Tau APFs in the brain and spinal cord of the P301L tauopathy mouse model. (a)** Tau APFs were detected by dot blots of brain tissue from P301L mice probed with αAPF, with a peak at 10 months. **(b)** Tau APFs were detected in the brainstem of P301L mice with αAPF IHC. **(c)** Tau APFs were also detected in P301L brains by double immunofluorescence using Tau-5 (green) and αAPF (red). Double labeling using AT8 (green) and αAPF (red) revealed a lack of colocalization, indicating that tau APFs were not phosphorylated at epitopes Ser202/Thr205 or that the phospho epitopes are inaccessible. **(d)** Tau APFs in oligodendrocytes were confirmed by double labeling using MOG and αAPF. Tau APFs were detected in the neurons of P301L mouse brain by double labeling with NeuN and αAPF, but double labeling with GFAP and αAPF demonstrated that tau APFs were not found in astrocytes in P301L brains. **(e)** Tau APFs were detected in 10-month-old P301L spinal cord by double immunofluorescence using Tau-5 (green) and αAPF (red). A low level of colocalization indicates that tau APFs are sporadically phosphorylated at epitopes Ser202/Thr205, specifically in axonal spheroids in the spinal cord. **(f)** Double labeling with GFAP (green) and αAPF (red) demonstrates that tau APFs are formed in astrocytes in P301L spinal cord. Tau APFs were also detected in neurons in P301L spinal cord. This was confirmed by double staining with NeuN (green) and αAPF (red). The location of tau APFs in oligodendrocytes was confirmed by double labeling for MOG (green) and αAPF (red). The scale bars for HRP **(b)** and IF **(c-f)** corresponded to 10 and 20 μm, respectively. White arrows indicated colocalization.

We next investigated whether APFs are present in the JNPL3 mouse model of tauopathy [39], which expresses the frontotemporal lobar degeneration-associated human mutant tau transgene P301L under the prion promoter. Mouse brain tissues were collected at different ages (2, 4, 7, 10, and 12 months) and biochemically analyzed for the presence of tau APFs, which were found to increase in an age-dependent fashion and peak at 10 months (Figure 5a). This finding is in accordance with a publication that reported that nearly 90% of JNPL3 mice develop motor and behavioral disturbances at 10 months old [39]. The decrease in tau APF levels in older mice (12 months) could be explained by the cellular loss that occurs in the model at that age. JNPL3 brain sections were then used in αAPF IHC experiments, which revealed APFs in the brainstem (Figure 5b). To verify that APF were comprised of tau protein, JNPL3 brain sections were double labeled with Tau-5 and αAPF. The results revealed that APFs colocalized with Tau-5 (Figure 5c). Conversely, no colocalization was observed following double labeling with AT8 and αAPF, demonstrating that tau APFs are not phosphorylated at epitopes Ser202/Thr205 (Figure 5c), similarly to human brain sections from patients with DLB. Next, cell specificity was examined by immunofluorescently labeling mouse brain sections. Tau APFs were detected in oligodendrocytes and neurons but not astrocytes (Figure 5d).Because severe tau pathology has been described in the JNPL3 spinal cord, we performed pathology studies to determine the presence of tau annular structures in this region. APFs detected were highly colocalized with Tau-5 (Figure 5e), affirming that tau APFs are present in the spinal cord. Double labeling with AT8 and αAPF revealed that tau APFs are sporadically phosphorylated at epitopes Ser202/Thr205 (Figure 5e). The annular structures detected with AT8 were identified as axonal spheroids, which may indicate disrupted axonal transport and degeneration. The presence of tau APFs phosphorylated at Ser202/Thr205 in the spinal cord suggests that annular structure formation occurs by a different mechanism than in the brain. To clarify which cell types are affected in the spinal cord, we performed double labeling with αAPF and GFAP, NeuN, or MOG (Figure 5f). Tau APFs were detected in all cell types analyzed (oligodendrocytes, neurons, and astrocytes), demonstrating that these tau structures are more prone to form in the spinal cord than in the brain.

Because tau APFs were mainly detected in oligodendrocytes in DLB and PSP patient brains and JNPL3 mouse brain and spinal cord, we studied the ability of MBP to induce the formation of APFs from tau oligomers. We incubated tau oligomers with MBP and performed kinetic analyses of APF formation with ELISAs using the αAPF antibody (Additional file 6: Figure S6a). The results show that mixing MBP with tau oligomers induces tau APF formation as early as 30 min after starting the incubation. Samples were also analyzed by AFM, which detected APFs after 3 h of incubation (Additional file 6: Figure S6b). No APFs were detected when Aβ oligomers were mixed with MBP or tau was mixed with lactalbumin, suggesting that this MBP-triggered process is specific for tau.

## Discussion

**Figure 6:**
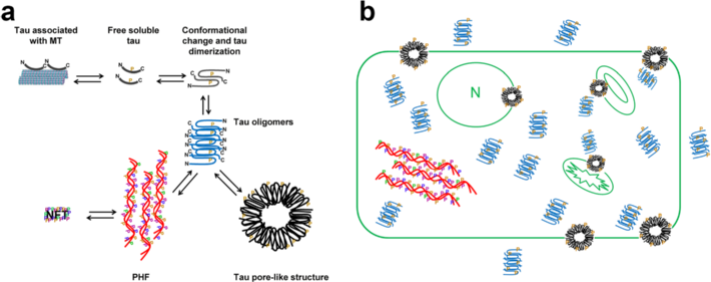
**Working model for tau APF formation. (a)** In the proposed mechanism for tau APF formation, tau associated with microtubules (MT) must be released to create an available pool of free soluble tau. Tau then undergoes a conformational change that facilitates misfolding and the formation of oligomers, which can then either proceed along the pathway to form paired helical filaments (PHF) that aggregate and form neurofibrillary tangles (NFTs) or align to form spherical, pore-like structures (APFs). **(b)** At the cellular level, the formation of these annular structures could occur whenever oligomers are associated in a suitable environment, such as the cell, nuclear, or organelle membranes, allowing for toxic, nonspecific ion leakage.

Amyloid-related diseases may be characterized by a common mechanism of aggregation and cytotoxicity that critically involves membrane permeabilization [30, 44, 45]. Several models have hypothesized how oligomers might disrupt cellular membranes, including transmembrane oligomeric pore structures reminiscent of those of pore-forming toxins [30, 45], nonspecific binding of amyloid oligomers to the membrane surface [44, 46], and detergent-like membrane dissolution by amyloid fibrils growing on the membrane surface [47]. As we previously reported for Aβ [28], tau oligomers are both the intermediates of tau fibril aggregation and the precursors of APF formation. The findings of the present study suggest that in vitro and in vivo tau APFs form via a pathway that is independent of NFT formation (Figure 6a). The mechanism of membrane-catalyzed conformational conversion of oligomers into annular pores is not yet well understood. It is possible that following an initial electrostatic interaction with the membrane, individual spherical oligomers are drawn into the core of the lipid bilayer where they undergo a conformational transition to expose their hydrophobic segments and assemble into pore-like structures [21, 48–51]. A similar membrane-catalyzed assembly has been proposed for the pore-forming toxins, in which the stepwise binding of the toxin to the membrane, membrane- catalyzed conformational change, and toxin oligomerization lead to the formation of membrane-embedded pores [32].

To our knowledge, this is the first study to describe the formation of tau APFs both in vitro and in vivo. However, the association of tau with the plasma membrane has been extensively described [52–54]. The authors of one study demonstrated a phosphorylation-dependent dynamic association between tau and the neuronal membrane [53]. Specifically, they showed that that dephosphorylation of tau at epitopes (including Ser202/Thr205) results in its translocation from the cytosol to the membrane, and this process is dependent on a direct interaction between Fyn and tau via the fyn-SH3 domain [55]. The function of plasma membrane-associated tau remains unclear; nevertheless, accumulating evidence suggests that the interaction between tau and Fyn might be crucial for the tau-mediated regulation of intracellular signaling [56]. Bhaskar and colleagues demonstrated that the affinity between Fyn and tau was increased 25–45 fold by the tau P301L mutation [55], which is the same mutation in the JNPL3 mouse model used in our study. This could explain the high amount of tau APFs detected in the JNPL3 model. Moreover, a recent study by Flach et al. revealed that tau oligomers impair artificial membrane integrity and cellular viability [57]. The authors showed that tau oligomers produce a deleterious effect on membrane permeabilization in SH-SY5Y cells. Furthermore, they corroborated these observations with a vesicle permeabilization assay, where they measured leakage of the fluorescent dye ANTS out of phospholipid vesicles. The authors observed a more pronounced effect in vesicles treated with tau oligomers rather than monomers or fibrils, suggesting that the cellular membrane might be a primary target of tau oligomers. Collectively, the existing evidence suggests that tau oligomers that are not phosphorylated at Ser202/Thr204 [40] could interact with Fyn and be taken into the plasma membrane given a suitable environment for tau APF formation. On the other hand, if tau oligomers continue their aggregation process and become phosphorylated at Ser202/Thr205, no interaction with Fyn will occur, leading to NFT formation. Further studies are necessary to establish the direct relationship between Fyn and tau APF formation.

EM analysis of pores isolated from human brains with AD and MSA inclusions revealed that they are indeed composed of individual spherical oligomers that vary in shape and size, lending support to the notion of nonspecific pore formation and membrane leakage [28, 58]. Nevertheless, other laboratories reported the ability of oligomers to form calcium channels in lipid bilayers and neuronal cells [59–61]. A study from Jang et al. [62] reported the ability of Aβ oligomers to assemble into pores with single-channel conductance that allow calcium uptake. Our results do not rule out the possibility of channel activity by tau pores. Either through the formation of channels or through nonspecific ion leakage, tau oligomers and APFs may lead to an influx of calcium and its subsequent dysregulation. NMDA receptor antagonists, such as memantine, block extrasynaptic calcium release and have been shown to be neuroprotective [63]. Recently, Kokubo et al. [35] reported that APFs were found not only on plasma membranes but also on vesicles inside of cell processes by immuno-EM using an αAPF antibody in transgenic mice expressing a mutant form of amyloid precursor protein [64]. This evidence suggests that several cellular environments can promote the formation of these annular structures. This last point was confirmed by our results showing tau APFs in different subcellular localizations, indicating that tau APFs are formed in both plasma and organelle membranes (Figure 6b). The present study also demonstrated that tau APFs are located in different cell types in DLB and PSP brains and JPNL3 brain sections and spinal cord. It is possible that the cell type in which tau APFs are formed will condition the clinical symptoms in different tauopathies or behavioral impairments in tau transgenic mouse models. Nevertheless, it is important to consider that P301L tau in the JNPL3 mouse model is expressed under the prion promoter, which is mainly expressed in neurons but also in oligodendrocytes and astrocytes. This is extremely important considering that it is well-known that tau is not expressed in astrocytes, suggesting different sources of tau in the formation of APFs in human tauopathies and the JNPL3 mouse model. While the presence of tau APFs in JPNL3 astrocytes could merely be due to the expression of tau in these cells, in the case of human tauopathies, the presence of these tau structures would be better explained by the internalization of exogenous tau into the astrocytes, which was previously suggested for Aβ and α-synuclein [29, 65].

We observed tau pores in oligodendrocytes in all of the studied samples. Several publications have demonstrated the relevance of tau aggregation in oligodendrocytes in different tauopathies and many mouse models [39, 42, 66–68]. Furthermore, a study by Klein et al. showed that Fyn interacts with tau in oligodendrocytes to promote myelination [69]. Specifically, the authors demonstrated that Fyn activation in lipid raft microdomains leads to an increased binding to tau and its recruitment to the area of contact with the neuronal axon. This concept is also supported by our in vitro data showing that MBP induces the formation of tau APFs, suggesting that areas rich in MBP are more prone to induce tau APF formation. The ability of MBP to induce the formation of annular structures from amyloid oligomers appears to be sequence-specific and is not a general mechanism for all amyloidogenic proteins (e.g., Aβ), which fails to form APFs in the presence of MBP. Nevertheless, previous studies have demonstrated that MBP binds to Aβ and inhibits amyloid fibril formation [70–72], suggesting that MBP does have some influence on the aggregation process of other amyloidogenic proteins.

Our novel findings linking tau oligomers with tau APFs enhance the understanding of amyloid oligomer-mediated neurodegeneration in vivo and underscore the complexity of tau structures in several tauopathies. Understanding the fundamentals of in vivo tau pore formation and properties is critical for establishing potential links between tau aggregation and the mechanisms of cellular toxicity that occur during neurodegenerative disease progression. Still, additional research should examine the nature of these tau pores and determine whether they represent single channels or nonspecific pores and clarify their relationship with Fyn.

## Electronic supplementary material

Additional file 1: Figure S1: Tau oligomers for APFs in vitro and in cell culture. (a) AFM was performed to observe how liposomes catalyze the conversion of oligomers to APFs. White arrowheads indicate tau APF formation. Scale bar, 10 nm. (b) Competitive ELISA showed that pre-incubation αAPF with tau APFs (4:1) led to the loss of the antibody’s ability to detect coated tau APFs. However, no effect was observed in the selectivity of αAPF for coated tau APFs when the antibody was pre-incubated with monomeric, oligomeric, or fibrillar tau (4:1). (c) Direct ELISA showed that T22 exclusively recognizes tau oligomers but not monomers, APFs, or fibrils. Tau-5 signals were equivalent when plates were coated with equal amounts of various tau species. (d) Competitive ELISA showed that when pre-incubation of T22 with tau oligomers (4:1) resulted in the loss of the antibody’s ability to detect coated tau oligomers. No effect on T22 selectivity for coated tau oligomers was observed when the antibody was pre-incubated with monomeric tau, APFs, or fibrillar tau (4:1). (e) Cytotoxicity was measured in SH-SY5Y cells using the MTT assay with 2 μM tau monomer, oligomer, APF or fibril. Tau oligomers were significantly more toxic than tau APFs. (f) Double immunofluorescence using Tau-5 (green) and αAPF (red) revealed tau APF formation when cells were treated with tau oligomers. White arrowheads indicate APFs. Scale bar, 10 μm. n = 6 per condition in b-e, *p < 0.05 and **p < 0.001. (TIFF 836 KB)

Additional file 2: Figure S2: Tau APFs are not phosphorylated at Ser202/Thr205. (a) Tau APFs were detected by double immunofluorescence using Tau-5 (green) and αAPF (red) antibodies in DLB and PSP sections. (b) In double labeling with AT8 (green) and αAPF (red), the lack of colocalization in all cases indicates that tau APFs are largely unphosphorylated at epitopes Ser202/Thr205. Scale bar, 10 μm. (TIFF 2 MB)

Additional file 3: Figure S3: Interaction between oligomers and APFs in DLB brains. Double labeling with the anti-tau oligomer antibody T22 (green) and the αAPF antibody (red) demonstrates an interaction between tau oligomers and APFs in vivo. (TIFF 620 KB)

Additional file 4: Figure S4: Cell specificity of tau APFs in PSP. Double labeling with GFAP (green) and αAPF (red) antibodies demonstrates that tau APFs are found in astrocytes in DLB brains. The presence of tau APFs in oligodendrocytes was confirmed by double labeling using MOG (green) and αAPF (red) antibodies. Tau APFs were also detected in neurons in DLB brains. This was confirmed by double staining using NeuN (green) and αAPF (red). Scale bar 10 μm. (TIFF 3 MB)

Additional file 5: Figure S5: Annular protofibrils from Aβ and α-synuclein were not detected in DLB cases. The lack of colocalization between αAPF (red) and 4G8 (green; top panels) or 4D6 (green; bottom panels) labeling suggests that Aβ APFs and α-synuclein APFs, respectively, are not present in DLB brains. (TIFF 11 MB)

Additional file 6: Figure S6: MBP induce tau APF formation. (a) Incubation of tau oligomers with MBP induced the formation of tau APFs as measured by ELISA using the αAPF antibody. MBP did not induce the formation of Aβ APFs from Aβ oligomers. Incubation of tau oligomers with albumin also failed to induce tau APF formation. (b) Tau APFs formed due to the interaction between oligomers and MBP were visualized by AFM. Scale bar, 5 nm. (TIFF 3 MB)

## References

[1] Binder LI, Frankfurter A, Rebhun LI (1985). The distribution of tau in the mammalian central nervous system. J Cell Biol.

[2] Drechsel DN, Hyman AA, Cobb MH, Kirschner MW (1992). Modulation of the dynamic instability of tubulin assembly by the microtubule-associated protein tau. Mol Biol Cell.

[3] Lee VM, Goedert M, Trojanowski JQ (2001). Neurodegenerative tauopathies. Annu Rev Neurosci.

[4] Alonso AC, Li B, Grundke-Iqbal I, Iqbal K (2008). Mechanism of tau-induced neurodegeneration in Alzheimer disease and related tauopathies. Curr Alzheimer Res.

[5] Iqbal K, Liu F, Gong CX, Alonso Adel C, Grundke-Iqbal I (2009). Mechanisms of tau-induced neurodegeneration. Acta Neuropathol.

[6] Ballatore C, Lee VM, Trojanowski JQ (2007). Tau-mediated neurodegeneration in Alzheimer’s disease and related disorders. Nat Rev Neurosci.

[7] Haroutunian V, Davies P, Vianna C, Buxbaum JD, Purohit DP (2007). Tau protein abnormalities associated with the progression of alzheimer disease type dementia. Neurobiol Aging.

[8] Braak H, Braak E (1991). Neuropathological stageing of Alzheimer-related changes. Acta Neuropathol.

[9] Delacourte A, Buee L (2000). Tau pathology: a marker of neurodegenerative disorders. Curr Opin Neurol.

[10] Braak H, Alafuzoff I, Arzberger T, Kretzschmar H, Del Tredici K (2006). Staging of Alzheimer disease-associated neurofibrillary pathology using paraffin sections and immunocytochemistry. Acta Neuropathol.

[11] Brunden KR, Trojanowski JQ, Lee VM (2008). Evidence that non-fibrillar tau causes pathology linked to neurodegeneration and behavioral impairments. J Alzheimers Dis.

[12] Marx J (2007). Alzheimer’s disease. A new take on tau. Science.

[13] Andorfer C, Acker CM, Kress Y, Hof PR, Duff K, Davies P (2005). Cell-cycle reentry and cell death in transgenic mice expressing nonmutant human tau isoforms. J Neurosci.

[14] Polydoro M, Acker CM, Duff K, Castillo PE, Davies P (2009). Age-dependent impairment of cognitive and synaptic function in the htau mouse model of tau pathology. J Neurosci.

[15] Yoshiyama Y, Higuchi M, Zhang B, Huang SM, Iwata N, Saido TC, Maeda J, Suhara T, Trojanowski JQ, Lee VM (2007). Synapse loss and microglial activation precede tangles in a P301S tauopathy mouse model. Neuron.

[16] Berger Z, Roder H, Hanna A, Carlson A, Rangachari V, Yue M, Wszolek Z, Ashe K, Knight J, Dickson D, Andorfer C, Rosenberry TL, Lewis J, Hutton M, Janus C (2007). Accumulation of pathological tau species and memory loss in a conditional model of tauopathy. J Neurosci.

[17] Spires TL, Orne JD, SantaCruz K, Pitstick R, Carlson GA, Ashe KH, Hyman BT (2006). Region-specific dissociation of neuronal loss and neurofibrillary pathology in a mouse model of tauopathy. Am J Pathol.

[18] Haass C, Selkoe DJ (2007). Soluble protein oligomers in neurodegeneration: lessons from the Alzheimer’s amyloid beta-peptide. Nat Rev Mol Cell Biol.

[19] Baglioni S, Casamenti F, Bucciantini M, Luheshi LM, Taddei N, Chiti F, Dobson CM, Stefani M (2006). Prefibrillar amyloid aggregates could be generic toxins in higher organisms. J Neurosci.

[20] Ross CA, Poirier MA (2005). Opinion: what is the role of protein aggregation in neurodegeneration?. Nat Rev Mol Cell Biol.

[21] Caughey B, Lansbury PT (2003). Protofibrils, pores, fibrils, and neurodegeneration: separating the responsible protein aggregates from the innocent bystanders. Annu Rev Neurosci.

[22] Glabe CG (2006). Common mechanisms of amyloid oligomer pathogenesis in degenerative disease. Neurobiol Aging.

[23] Miller Y, Ma B, Nussinov R (2011). Synergistic interactions between repeats in tau protein and Abeta amyloids may be responsible for accelerated aggregation via polymorphic states. Biochemistry.

[24] Hafner JH, Cheung CL, Woolley AT, Lieber CM (2001). Structural and functional imaging with carbon nanotube AFM probes. Prog Biophys Mol Biol.

[25] Ding TT, Lee SJ, Rochet JC, Lansbury PT (2002). Annular alpha-synuclein protofibrils are produced when spherical protofibrils are incubated in solution or bound to brain-derived membranes. Biochemistry.

[26] Lashuel HA, Hartley D, Petre BM, Walz T, Lansbury PT (2002). Neurodegenerative disease: amyloid pores from pathogenic mutations. Nature.

[27] Pountney DL, Lowe R, Quilty M, Vickers JC, Voelcker NH, Gai WP (2004). Annular alpha-synuclein species from purified multiple system atrophy inclusions. J Neurochem.

[28] Lasagna-Reeves CA, Glabe CG, Kayed R (2011). Amyloid-beta annular protofibrils evade fibrillar fate in Alzheimer disease brain. J Biol Chem.

[29] Lasagna-Reeves CA, Kayed R (2011). Astrocytes contain amyloid-beta annular protofibrils in Alzheimer’s disease brains. FEBS Lett.

[30] Lashuel HA, Lansbury PT (2006). Are amyloid diseases caused by protein aggregates that mimic bacterial pore-forming toxins?. Q Rev Biophys.

[31] Kayed R, Pensalfini A, Margol L, Sokolov Y, Sarsoza F, Head E, Hall J, Glabe C (2009). Annular protofibrils are a structurally and functionally distinct type of amyloid oligomer. J Biol Chem.

[32] Montoya M, Gouaux E (2003). Beta-barrel membrane protein folding and structure viewed through the lens of alpha-hemolysin. Biochim Biophys Acta.

[33] Parker MW, Feil SC (2005). Pore-forming protein toxins: from structure to function. Prog Biophys Mol Biol.

[34] Glabe CG, Kayed R (2006). Common structure and toxic function of amyloid oligomers implies a common mechanism of pathogenesis. Neurology.

[35] Kokubo H, Kayed R, Glabe CG, Staufenbiel M, Saido TC, Iwata N, Yamaguchi H (2009). Amyloid Beta annular protofibrils in cell processes and synapses accumulate with aging and Alzheimer-associated genetic modification. Int J Alzheimers Dis.

[36] Margittai M, Langen R (2004). Template-assisted filament growth by parallel stacking of tau. Proc Natl Acad Sci USA.

[37] Lasagna-Reeves CA, Castillo-Carranza DL, Guerrero-Muoz MJ, Jackson GR, Kayed R (2010). Preparation and characterization of neurotoxic tau oligomers. Biochemistry.

[38] Lasagna-Reeves CA, Castillo-Carranza DL, Sengupta U, Clos AL, Jackson GR, Kayed R (2011). Tau oligomers impair memory and induce synaptic and mitochondrial dysfunction in wild-type mice. Mol Neurodegener.

[39] Lewis J, McGowan E, Rockwood J, Melrose H, Nacharaju P, Van Slegtenhorst M, Gwinn-Hardy K, Paul Murphy M, Baker M, Yu X, Duff K, Hardy J, Corral A, Lin WL, Yen SH, Dickson DW, Davies P, Hutton M (2000). Neurofibrillary tangles, amyotrophy and progressive motor disturbance in mice expressing mutant (P301L) tau protein. Nat Genet.

[40] Lasagna-Reeves CA, Castillo-Carranza DL, Sengupta U, Sarmiento J, Troncoso J, Jackson GR, Kayed R (2012). Identification of oligomers at early stages of tau aggregation in Alzheimer’s disease. FASEB J.

[41] Iseki E, Togo T, Suzuki K, Katsuse O, Marui W, de Silva R, Lees A, Yamamoto T, Kosaka K (2003). Dementia with Lewy bodies from the perspective of tauopathy. Acta Neuropathol.

[42] Yoshida M (2006). Cellular tau pathology and immunohistochemical study of tau isoforms in sporadic tauopathies. Neuropathology.

[43] Augustinack JC, Schneider A, Mandelkow EM, Hyman BT (2002). Specific tau phosphorylation sites correlate with severity of neuronal cytopathology in Alzheimer’s disease. Acta Neuropathol.

[44] Sokolov Y, Kozak JA, Kayed R, Chanturiya A, Glabe C, Hall JE (2006). Soluble amyloid oligomers increase bilayer conductance by altering dielectric structure. J Gen Physiol.

[45] Quist A, Doudevski I, Lin H, Azimova R, Ng D, Frangione B, Kagan B, Ghiso J, Lal R (2005). Amyloid ion channels: a common structural link for protein-misfolding disease. Proc Natl Acad Sci USA.

[46] Kayed R, Sokolov Y, Edmonds B, McIntire TM, Milton SC, Hall JE, Glabe CG (2004). Permeabilization of lipid bilayers is a common conformation-dependent activity of soluble amyloid oligomers in protein misfolding diseases. J Biol Chem.

[47] Engel MF, Khemtemourian L, Kleijer CC, Meeldijk HJ, Jacobs J, Verkleij AJ, de Kruijff B, Killian JA, Hoppener JW (2008). Membrane damage by human islet amyloid polypeptide through fibril growth at the membrane. Proc Natl Acad Sci USA.

[48] Zhu M, Li J, Fink AL (2003). The association of alpha-synuclein with membranes affects bilayer structure, stability, and fibril formation. J Biol Chem.

[49] Chen F-Y, Lee M-T, Huang HW (2003). Evidence for membrane thinning effect as the mechanism for peptide-induced pore formation. Biophys J.

[50] Modler AJ, Gast K, Lutsch G, Damaschun G (2003). Assembly of amyloid protofibrils via critical oligomers–a novel pathway of amyloid formation. J Mol Biol.

[51] Porat Y, Kolusheva S, Jelinek R, Gazit E (2003). The human islet amyloid polypeptide forms transient membrane-active prefibrillar assemblies. Biochemistry.

[52] Pooler AM, Hanger DP (2010). Functional implications of the association of tau with the plasma membrane. Biochem Soc Trans.

[53] Pooler AM, Usardi A, Evans CJ, Philpott KL, Noble W, Hanger DP (2012). Dynamic association of tau with neuronal membranes is regulated by phosphorylation. Neurobiol Aging.

[54] Maas T, Eidenmuller J, Brandt R (2000). Interaction of tau with the neural membrane cortex is regulated by phosphorylation at sites that are modified in paired helical filaments. J Biol Chem.

[55] Bhaskar K, Yen SH, Lee G (2005). Disease-related modifications in tau affect the interaction between Fyn and Tau. J Biol Chem.

[56] Ittner LM, Ke YD, Delerue F, Bi M, Gladbach A, van Eersel J, Wolfing H, Chieng BC, Christie MJ, Napier IA, Eckert A, Staufenbiel M, Hardeman E, Gotz J (2010). Dendritic function of tau mediates amyloid-beta toxicity in Alzheimer’s disease mouse models. Cell.

[57] Flach K, Hilbrich I, Schiffmann A, Gartner U, Kruger M, Leonhardt M, Waschipky H, Wick L, Arendt T, Holzer M (2012). Tau oligomers impair artificial membrane integrity and cellular viability. J Biol Chem.

[58] Pountney DL, Lowe R, Quilty M, Vickers JC, Voelcker NH, Gai WP (2004). Annular alpha-synuclein species from purified multiple system atrophy inclusions. J Neurochem.

[59] Arispe N, Pollard HB, Rojas E (1993). Giant multilevel cation channels formed by Alzheimer disease amyloid beta-protein [A beta P-(1–40)] in bilayer membranes. Proc Natl Acad Sci USA.

[60] Kagan BL, Hirakura Y, Azimov R, Azimova R, Lin MC (2002). The channel hypothesis of Alzheimer’s disease: current status. Peptides.

[61] Di Scala C, Troadec J-D, Lelièvre C, Garmy N, Fantini J, Chahinian H (2013). Mechanism of cholesterol-assisted oligomeric channel formation by a short Alzheimer β-amyloid peptide. J Neurochem.

[62] Jang H, Arce FT, Ramachandran S, Capone R, Azimova R, Kagan BL, Nussinov R, Lal R (2010). Truncated beta-amyloid peptide channels provide an alternative mechanism for Alzheimer’s disease and down syndrome. Proc Natl Acad Sci USA.

[63] Bordji K, Becerril-Ortega J, Buisson A (2011). Synapses, NMDA receptor activity and neuronal Aβ production in Alzheimer’s disease. Rev Neurosci.

[64] Yamaguchi H, Maat-Schieman ML, van Duinen SG, Prins FA, Neeskens P, Natte R, Roos RA (2000). Amyloid beta protein (Abeta) starts to deposit as plasma membrane-bound form in diffuse plaques of brains from hereditary cerebral hemorrhage with amyloidosis-Dutch type, Alzheimer disease and nondemented aged subjects. J Neuropathol Exp Neurol.

[65] Lee HJ, Suk JE, Patrick C, Bae EJ, Cho JH, Rho S, Hwang D, Masliah E, Lee SJ (2010). Direct transfer of alpha-synuclein from neuron to astroglia causes inflammatory responses in synucleinopathies. J Biol Chem.

[66] Nishimura M, Tomimoto H, Suenaga T, Namba Y, Ikeda K, Akiguchi I, Kimura J (1995). Immunocytochemical characterization of glial fibrillary tangles in Alzheimer’s disease brain. Am J Pathol.

[67] Allen B, Ingram E, Takao M, Smith MJ, Jakes R, Virdee K, Yoshida H, Holzer M, Craxton M, Emson PC, Atzori C, Migheli A, Crowther RA, Ghetti B, Spillantini MG, Goedert M (2002). Abundant tau filaments and nonapoptotic neurodegeneration in transgenic mice expressing human P301S tau protein. J Neurosci.

[68] Ren Y, Lin WL, Sanchez L, Ceballos C, Polydoro M, Spires-Jones TL, Hyman BT, Dickson DW, Sahara N (2013). Endogenous Tau Aggregates in Oligodendrocytes of rTg4510 Mice Induced by Human P301L Tau. J Alzheimers Dis.

[69] Klein C, Kramer EM, Cardine AM, Schraven B, Brandt R, Trotter J (2002). Process outgrowth of oligodendrocytes is promoted by interaction of fyn kinase with the cytoskeletal protein tau. J Neurosci.

[70] Hoos MD, Ahmed M, Smith SO, Van Nostrand WE (2009). Myelin basic protein binds to and inhibits the fibrillar assembly of Abeta42 in vitro. Biochemistry.

[71] Liao MC, Hoos MD, Aucoin D, Ahmed M, Davis J, Smith SO, Van Nostrand WE (2010). N-terminal domain of myelin basic protein inhibits amyloid beta-protein fibril assembly. J Biol Chem.

[72] Liao MC, Ahmed M, Smith SO, Van Nostrand WE (2009). Degradation of amyloid beta protein by purified myelin basic protein. J Biol Chem.

