# Supplementary figures and images for: The formation of tau pore-like structures is prevalent and cell specific: possible implications for the disease phenotypes
Source: Acta Neuropathol Commun. 2014 May 29;2:56. doi: 10.1186/2051-5960-2-56 (PMC4231072; doi:10.1186/2051-5960-2-56)

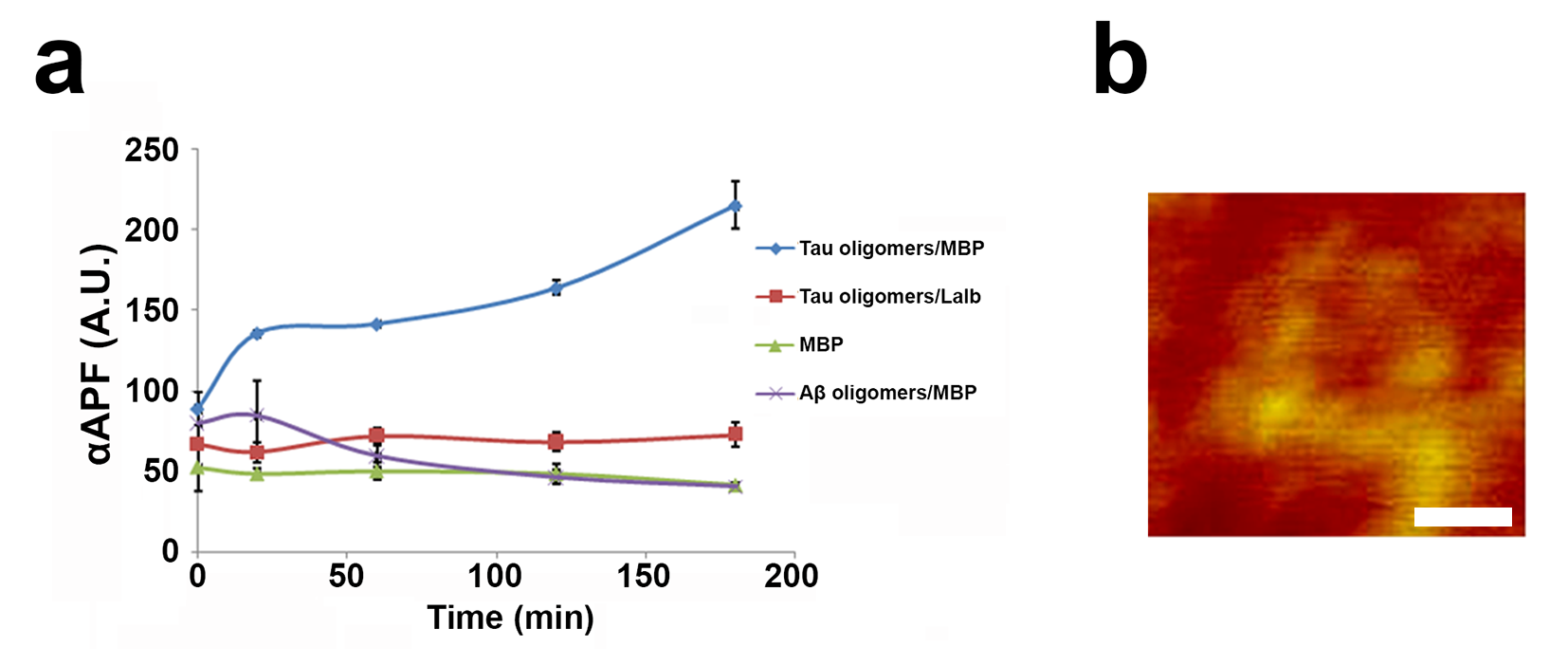

Supplement: Supplementary file 1 — Additional file 1: Figure S1: Tau oligomers for APFs in vitro and in cell culture. (a) AFM was performed to observe how liposomes catalyze the conversion of oligomers to APFs. White arrowheads indicate tau APF formation. Scale bar, 10 nm. (b) Competitive ELISA showed that pre-incubation αAPF with tau APFs (4:1) led to the loss of the antibody’s ability to detect coated tau APFs. However, no effect was observed in the selectivity of αAPF for coated tau APFs when the antibody was pre-incubated with monomeric, oligomeric, or fibrillar tau (4:1). (c) Direct ELISA showed that T22 exclusively recognizes tau oligomers but not monomers, APFs, or fibrils. Tau-5 signals were equivalent when plates were coated with equal amounts of various tau species. (d) Competitive ELISA showed that when pre-incubation of T22 with tau oligomers (4:1) resulted in the loss of the antibody’s ability to detect coated tau oligomers. No effect on T22 selectivity for coated tau oligomers was observed when the antibody was pre-incubated with monomeric tau, APFs, or fibrillar tau (4:1). (e) Cytotoxicity was measured in SH-SY5Y cells using the MTT assay with 2 μM tau monomer, oligomer, APF or fibril. Tau oligomers were significantly more toxic than tau APFs. (f) Double immunofluorescence using Tau-5 (green) and αAPF (red) revealed tau APF formation when cells were treated with tau oligomers. White arrowheads indicate APFs. Scale bar, 10 μm. n = 6 per condition in b-e, *p < 0.05 and **p < 0.001. (TIFF 836 KB) [file 40478_2014_131_MOESM1_ESM.tiff]

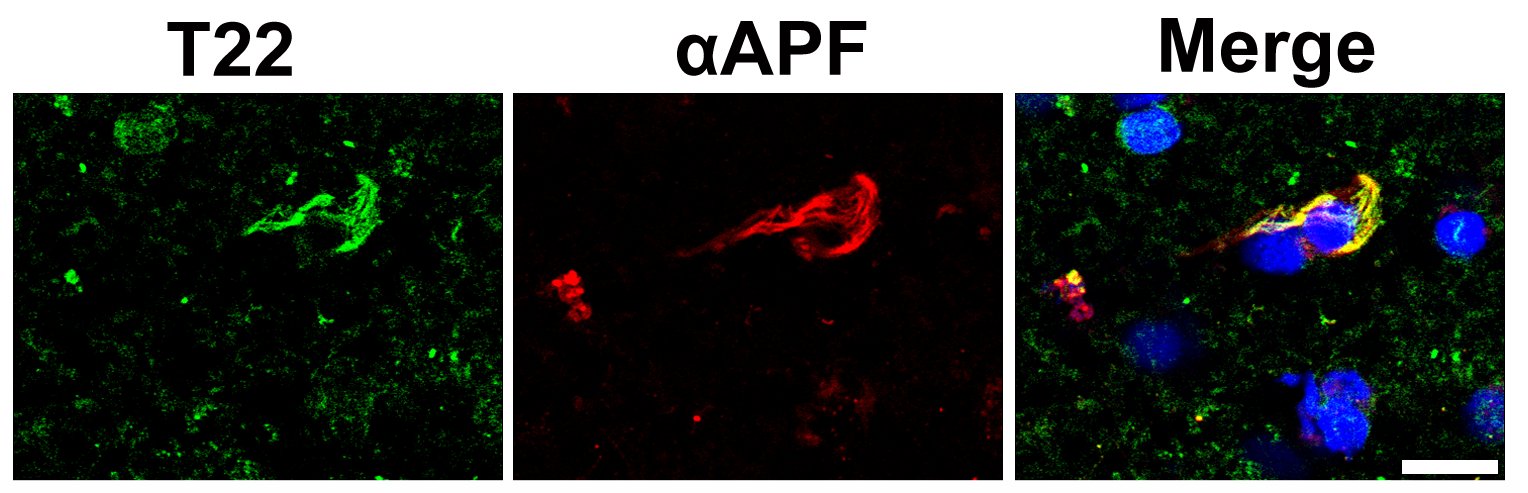

Supplement: Supplementary file 2 — Additional file 2: Figure S2: Tau APFs are not phosphorylated at Ser202/Thr205. (a) Tau APFs were detected by double immunofluorescence using Tau-5 (green) and αAPF (red) antibodies in DLB and PSP sections. (b) In double labeling with AT8 (green) and αAPF (red), the lack of colocalization in all cases indicates that tau APFs are largely unphosphorylated at epitopes Ser202/Thr205. Scale bar, 10 μm. (TIFF 2 MB) [file 40478_2014_131_MOESM2_ESM.tiff]

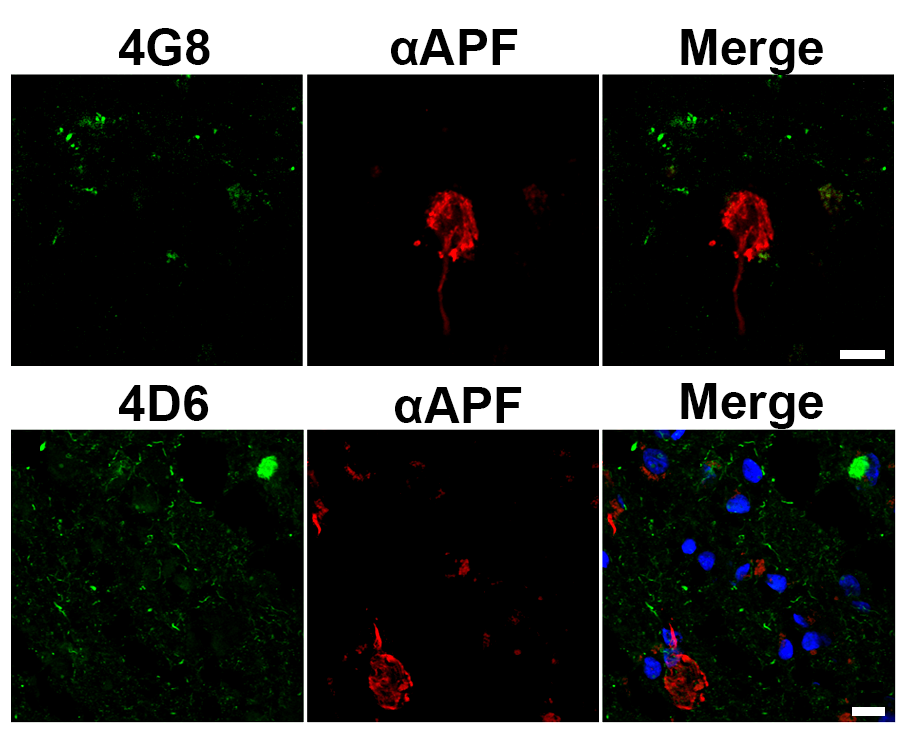

Supplement: Supplementary file 3 — Additional file 3: Figure S3: Interaction between oligomers and APFs in DLB brains. Double labeling with the anti-tau oligomer antibody T22 (green) and the αAPF antibody (red) demonstrates an interaction between tau oligomers and APFs in vivo. (TIFF 620 KB) [file 40478_2014_131_MOESM3_ESM.tiff]

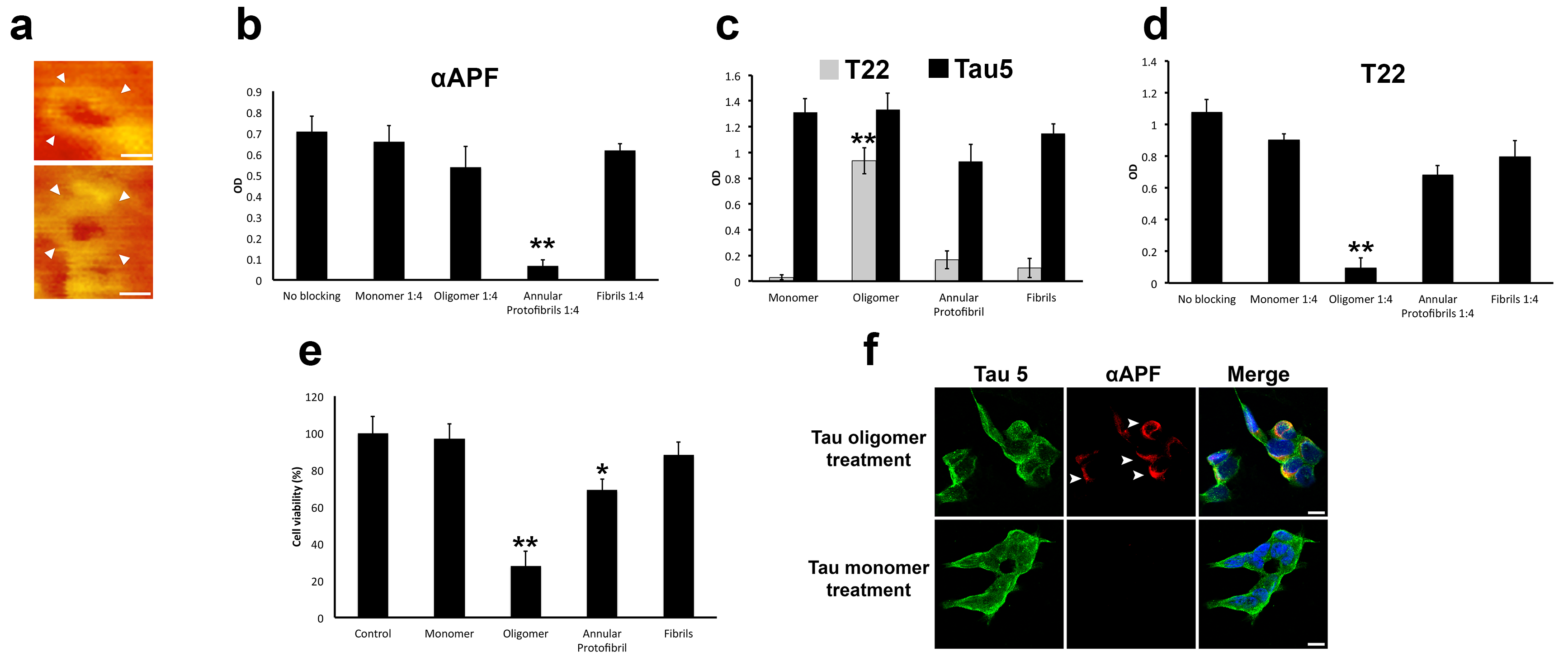

Supplement: Supplementary file 4 — Additional file 4: Figure S4: Cell specificity of tau APFs in PSP. Double labeling with GFAP (green) and αAPF (red) antibodies demonstrates that tau APFs are found in astrocytes in DLB brains. The presence of tau APFs in oligodendrocytes was confirmed by double labeling using MOG (green) and αAPF (red) antibodies. Tau APFs were also detected in neurons in DLB brains. This was confirmed by double staining using NeuN (green) and αAPF (red). Scale bar 10 μm. (TIFF 3 MB) [file 40478_2014_131_MOESM4_ESM.tiff]

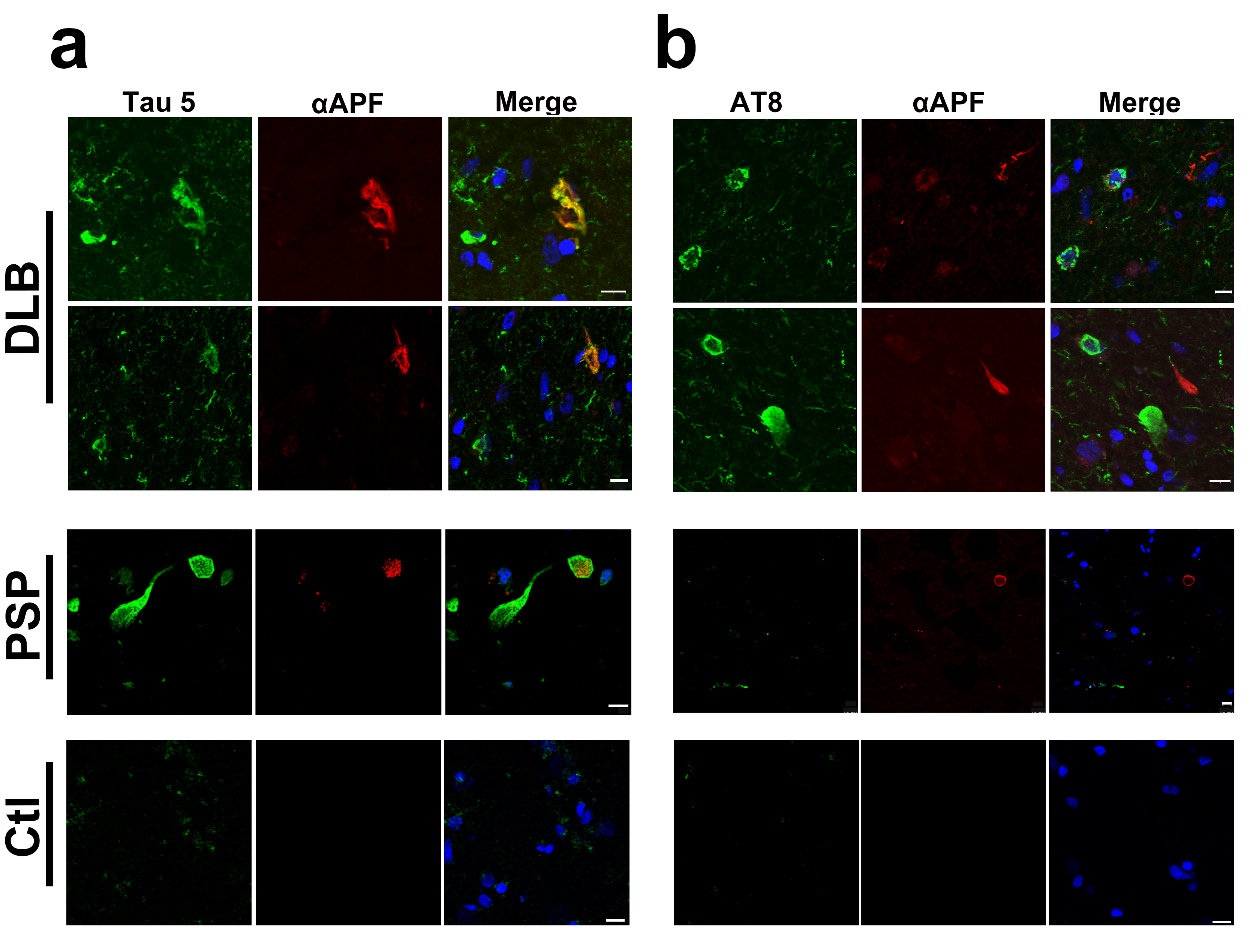

Supplement: Supplementary file 5 — Additional file 5: Figure S5: Annular protofibrils from Aβ and α-synuclein were not detected in DLB cases. The lack of colocalization between αAPF (red) and 4G8 (green; top panels) or 4D6 (green; bottom panels) labeling suggests that Aβ APFs and α-synuclein APFs, respectively, are not present in DLB brains. (TIFF 11 MB) [file 40478_2014_131_MOESM5_ESM.tiff]

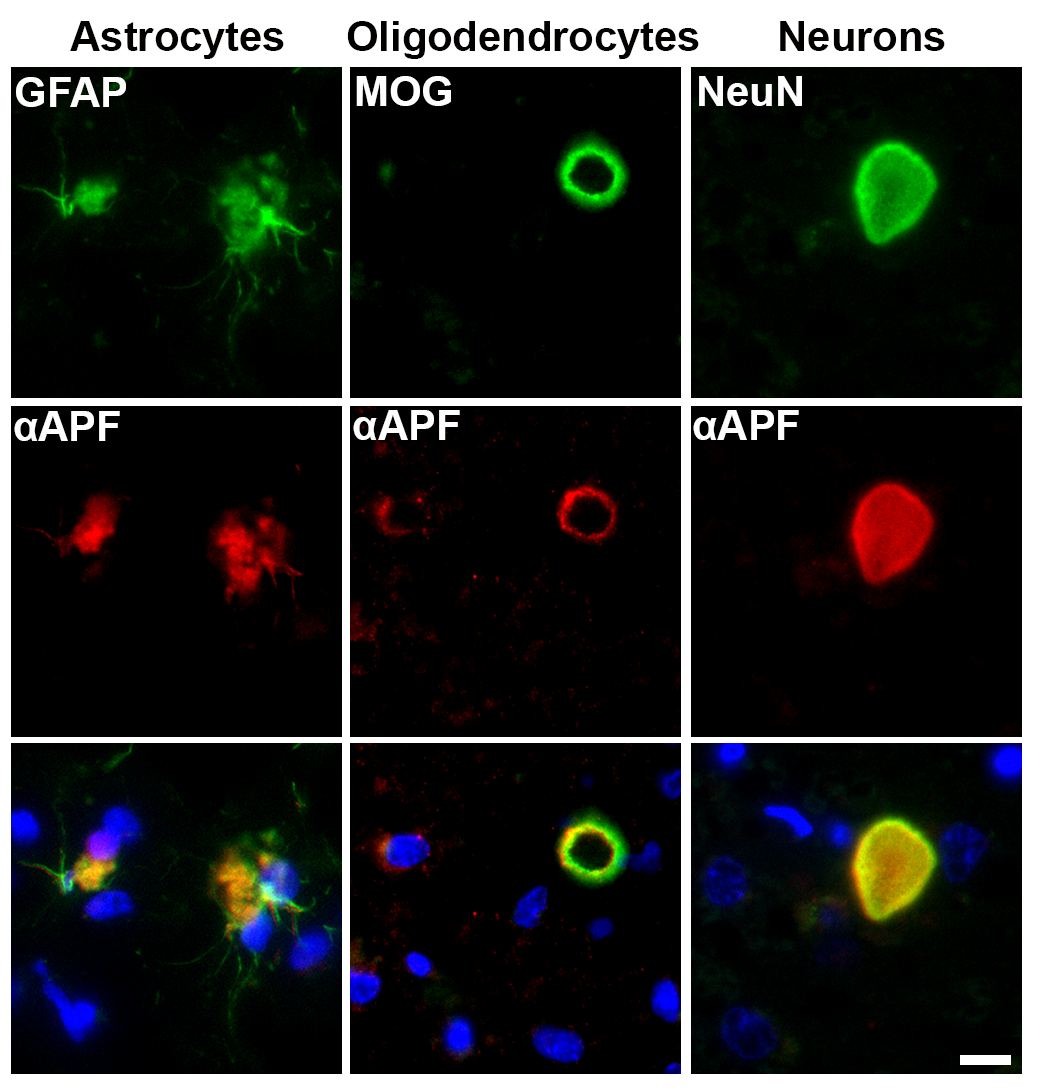

Supplement: Supplementary file 6 — Additional file 6: Figure S6: MBP induce tau APF formation. (a) Incubation of tau oligomers with MBP induced the formation of tau APFs as measured by ELISA using the αAPF antibody. MBP did not induce the formation of Aβ APFs from Aβ oligomers. Incubation of tau oligomers with albumin also failed to induce tau APF formation. (b) Tau APFs formed due to the interaction between oligomers and MBP were visualized by AFM. Scale bar, 5 nm. (TIFF 3 MB) [file 40478_2014_131_MOESM6_ESM.tiff]
